# Significance of duon mutations in cancer genomes

**DOI:** 10.1038/srep27437

**Published:** 2016-06-08

**Authors:** Vinod Kumar Yadav, Kyle S. Smith, Colin Flinders, Shannon M. Mumenthaler, Subhajyoti De

**Affiliations:** 1 Department of Medicine, University of Colorado School of Medicine, Aurora, CO 80045, USA; 2 The Jackson Laboratory, Farmington, CT06032, USA; 3 Computational Biosciences Graduate Program, University of Colorado, Aurora, CO 80045, USA; 4 Center for Applied Molecular Medicine, University of Southern California, Los Angeles, CA, 90033, USA; 5 University of Colorado Cancer Center, Aurora 80045, CO, USA; 6 Rutgers Cancer Institute of New Jersey, New Brunswick, NJ 08901, USA

## Abstract

Functional mutations in coding regions not only affect the structure and function of the protein products, but may also modulate their expression in some cases. This class of mutations, recently dubbed “duon mutations” due to their dual roles, can potentially have major impacts on downstream pathways. However their significance in diseases such as cancer remain unclear. In a survey covering 4606 samples from 19 cancer types, and integrating allelic expression, overall mRNA expression, regulatory motif perturbation, and chromatin signatures in one composite index called REDACT score, we identified potential duon mutations. Several such mutations are detected in known cancer genes in multiple cancer types. For instance a potential duon mutation in TP53 is associated with increased expression of the mutant allelic gene copy, thereby possibly amplifying the functional effects on the downstream pathways. Another potential duon mutation in SF3B1 is associated with abnormal splicing and changes in angiogenesis and matrix degradation related pathways. Our findings emphasize the need to interrogate the mutations in coding regions beyond their obvious effects on protein structures.

Symbolic complexity of DNA sequences allows encryption of more than one genetic code in the same sequence. While the concept is not new, emerging findings suggest that overlapping coding and regulatory codes might be more common in the human genome than previously anticipated. Stergachis *et al.*^1^ coined a term ‘duon’ to report a class of regulatory sequences within protein-coding regions, which not only code for and hence govern structure and/or function of the underlying RNA or protein sequences, but also control their regulation at the level of transcription or translation. By initial estimates, duons are widespread–~15% of the codons within 86.9% of human genes have regulatory potential consistent with duon-like function^1^,^2^.

There are several reported instances of mutations in regulatory elements within protein coding genes in cancer. For instance, HIF1A protein-level expression is regulated by oxygen-controlled ubiquitination that is disrupted by deletions and missense mutations^3^. There are other examples of regulatory mutations in coding regions. A recurrent, somatic, synonymous mutation (F17F) in BCL2L12 increases its expression by altering the binding site of miR-671-5p in melanoma, which in turn affects its interaction with TP53, inhibiting apoptosis^4^. This mutation is found in ~4% of melanoma cases^4^. Supek *et al.* presented a compelling finding where synonymous mutations alter transcript splicing, thereby affecting both protein function and regulation^5^. Taken together, these findings indicate that regulatory mutations in coding regions might be common in cancer. Interestingly, mutations in the duon elements can create a mutant gene copy and also modulate its expression in a single hit, thereby potentially amplifying (or moderating) its functional impacts on the downstream pathways (Fig. 1A). But the significance of the mutations, which create or perturb duon elements, denoted as ‘duon mutations’ from here after, in diseases such as cancer remains poorly understood.

## Results

**Figure 1:**
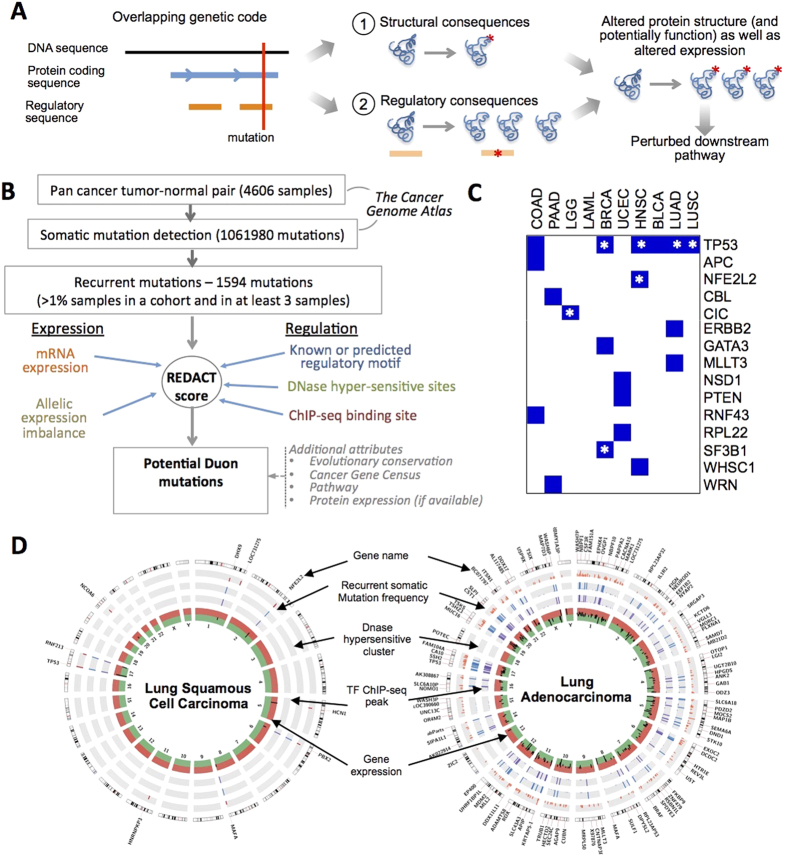
An overview of the potential duon mutations in human cancers. (**A**) A Schematic representation showing mutations in Duon elements in protein-coding regions, which have dual roles of regulating gene expression, besides coding for gene products. These mutations have the potential to perturb downstream pathways by altering both structure and expression of a gene product. (**B**) A schematic diagram showing the analysis pipeline. (**C**) The summary of potential Duon mutations in cancer genes; box with asterisk representing missense mutation. (**D**) Circos plot showing the genome-wide landscapes of recurrent coding mutations, including those that overlap with DNase hypersensitive cluster, transcription factor ChIP-seq peaks, and altered expression of the genes that harbor them, for both lung adenocarcinoma and squamous cell carcinoma. Only DNase regions, ChIP-seq peaks overlap with recurrent mutations were shown in the plot.

We conducted a survey covering 4606 samples from 19 cancer types, and identified recurrent, potential duon mutations after integrating mRNA and protein expression, allelic expression imbalance, epigenetic makeup, regulatory potential, and pathway data (Supplementary Table 1; Flow chart of analysis pipeline used in our approach presented as Supplementary Fig. 1). Our initial dataset included all the major, adult cancer types and had a total of 1,061,980 somatic, exonic point mutations and InDels (Fig. 1B and Supplementary Fig. 2).

### Criteria for selection and REDACT score

While the criteria for duon mutation are still evolving, here we define that a recurrent, potential duon mutation (pDM) would have at least the following attributes - (i) it is recurrently detected in one or more cancer cohorts, (ii) it alters protein sequence (e.g. missense mutations, which in turn could alter structure and/or function of the protein) (iii) it is associated with altered expression of the gene product in the affected samples, thereby having ‘dual’ effects, and (iv) it occurs in genomic regions with epigenetic makeups and sequence contexts consistent with regulatory activities. In this study we did not consider those duon mutations which could potentially alter expression of gene products by post-transcriptional or post translational modifications, as well as nonsense, frame-shift, and splice-site mutations.

We combined somatic mutations, mRNA and protein expression, allelic expression imbalance, and regulatory potential data to prioritize pDMs. We developed a scoring system, dubbed REDACT score, to identify recurrent pDMs and summarize their supporting evidence. The rationale behind the scoring system was three-fold. First, a pDM may not have all possible supporting data-types available. For instance, in our cohort, protein-level expression data was available for a selected set of proteins only. Second, a pDM may not have statistically significant signal at each level (even though the signal may be consistent across different levels), especially when the sample size is small (95% mutations present in <13 samples; Supplementary Fig. 2). Third, it is not always straightforward make inferences based on noisy signals from multiple heterogeneous data types.

Thus, we evaluated the pDMs for 6 types of evidence (i) ***R***ecurrence: only those mutations which were present in at least 3 samples as well as >1% samples in a cohort were considered, (ii) mRNA-level ***E***xpression change: the target genes were expected to show significantly different mRNA expression in affected samples compared to other samples in the cohort (p-value < 0.05) (iii) overlap with ***D***Nase hypersensitive regions: mutations that overlapped with DNase hypersensitive regions in reference tissue or cell lines were prioritized, (iv) ***A***llelic expression imbalance: mutant gene copy was expected to show systematic difference in allelic expression compared to the wild-type gene copy. Wherever possible, we combined tumor purity, exomeseq and RNAseq data to examine allelic expression imbalance of the mutant allele after adjusting for tumor purity and clonality. Majority of the pDMs showed systematic increase (or decrease) in expression of the mutant alleles relative to corresponding wild type alleles; furthermore these changes were also consistent with the overall increase (or decrease) in mRNA-level expression levels, (v) overlap with ***C***hromatin immune-precipitation (ChIP) peaks: mutations that overlapped with ChIP-seq or ChIP-chip peaks for transcription factors in the RegulomeDB^6^ were preferred, (vi) ***T***ranscription factor binding motif perturbation: mutations that contributed to creation or perturbation of predicted transcription factor binding motif based on their position weight matrix were considered. The 6-letter composite REDACT score summarized the supporting evidence at the levels described above. In addition, we calculated combined p-value from different lines of evidences (see Methods for detail). We focused on the mutations that are recurrent (R), associated with altered mRNA expression (E), and have at least one type of evidence available in support of regulatory potential (D, C, or T).

To demonstrate the utility of our approach, we selected rs8110393 (G > A) a nonsynonymous germ line SNP in RINL, which has been previously classified as a duon mutation^1^. We found that (i) it is recurrent with population allele frequency >1% in the 1000 Genomes Project cohort, (ii) it is a known eQTL variant, (iii) it overlaps with DNase hypersensitive region, ChIP peaks and TFBS motif. Therefore, extending our criteria, it could be classified as a germ line pDM with a full REDACT score (Supplementary Fig. 3).

### Assessment of potential duon mutations in cancer

In the analysis of 4606 samples from 19 cancer types we found 146 somatic mutations in 135 protein coding genes that were recurrent (R) and were associated with mRNA-level expression changes (E); of them 121 variants in 108 genes also had evidence for regulatory potential (D, C, or T) as well. Of them, 20 are missense mutations, and 30 were in-frame InDels, which we denote as potential duon mutations. Among these mutations, several were in known cancer genes (including TP53, SF3B1, APC and PTEN; Fig. 1C and Supplementary Table 2). A vast majority of those had significant combined p-value. Several pDMs were present in multiple cancer types. We discuss selected examples in greater details below.

### Missense pDM in TP53

**Figure 2:**
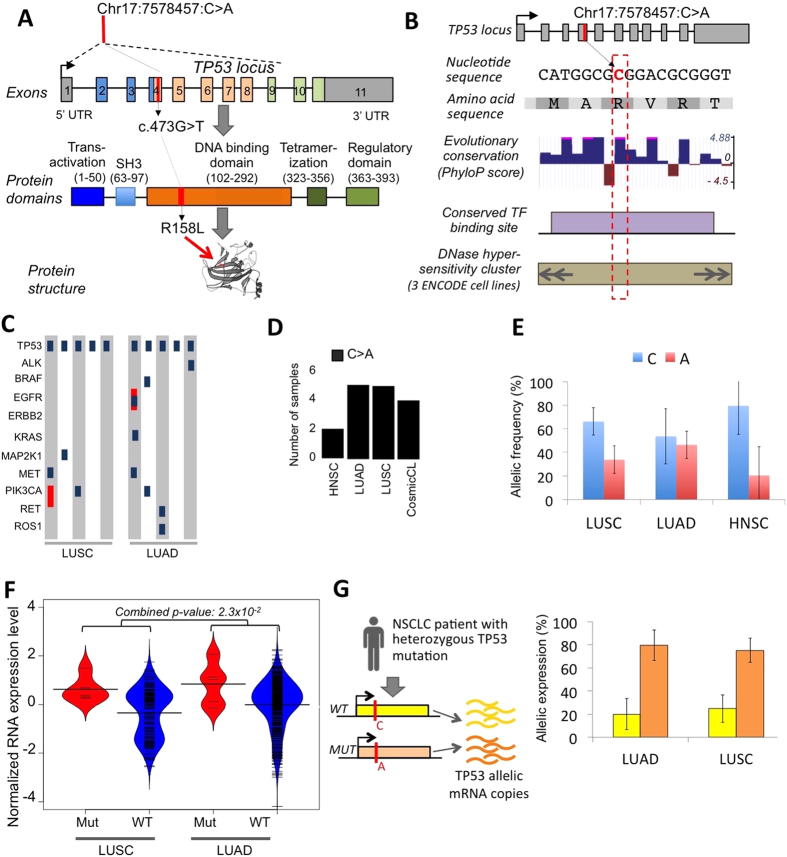
A somatic mutation with signatures of potential duon mutation in TP53. (**A**) Location of the pDM (Chr17:7578457:C > A) in the coding region of TP53 is shown. (**B**) Summary diagram showing base by base Phastcons evolutionary conservation, predicted transcription factor (E2F; HINFP1) binding motif, and overlap with DNase hypersensitivity sites in multiple ENCODE cell lines. (**C**) Actionable driver mutations in the samples that have the TP53 pDM in lung cancer. Blue: point mutation, red: amplification, green: deletion. (**D**) Presence of the pDM in different cancer types. (**E**) Allelic frequency estimates of the TP53 pDM in different cancer shows that it is predominantly clonal in majority of the cases. Plot represents mean allelic frequency per cancer type with 95% CI of mean as error bars. (**F**) Beanplot showing TP53 mRNA level expression of the samples carrying p.R158L pDM, compared to other samples that are wild type at that site for lung squamous cell carcinoma and adenocarcinoma. Statistical significance was estimated using Mann Whitney U test, and combined p-value estimated using Fisher’s method. (**G**) Allelic mRNA expression pattern at the site of mutation showed allelic imbalance: the A allele had relatively higher expression than the C allele in all the mutated samples. Plot represents mean allelic mRNA expression per cancer type with 95% CI of mean as error bars.

We found a recurrent missense mutation in TP53 (Chr17:7578457:C > A; p.R158L) in lung adenocarcinoma^7^ (5 samples) and lung squamous cell carcinoma^8^ (5 samples) with attributes consistent with the definition of pDM (Fig. 1D and Supplementary Table 3). This mutation in the DNA binding domain is known to affect TP53 structure and function (Fig. 2A)^9^,^10^,^11^ but its potential duon-like activity was not previously reported. The nucleotide-position had high evolutionary conservation, and also overlapped predicted regulatory motifs and DNase hypersensitive sites in multiple ENCODE cell lines (Fig. 2B). The mutation co-occurred with several other actionable driver mutations in the lung cancer samples (Fig. 2C). The position was mutated in multiple different cancer types, and the C > A substitution was detected in lung cancer, head and neck cancer patient samples, as well as cancer cell lines analyzed in the COSMIC project (Fig. 2D). In a majority of the affected samples the mutations were present at high allele frequencies (Fig. 2E), indicating that those were probably early mutation events.

Integrating RNAseq expression data for the lung cancer samples, we found that the affected samples had significantly high TP53 expression compared to other lung cancer samples in the cohort (p-value: 2.3E-02) (Fig. 2F). In addition, the mutant allele had significantly higher expression level compared to the wild-type allele in all the affected samples, which is evident after adjusting for tumor purity and clonality (see Methods; Binomial test with Fisher’s combined p-value < 0.001) (Fig. 2G). We additionally interrogated SNP array data for the same samples and found that the affected samples did not have detectable copy number alteration that could confound our observations (Supplementary Fig. 4). Furthermore, an increase in expression of the mutant TP53 copy was matched with a corresponding decrease in the expression of the TP53 wild type copy, perhaps due to transcriptional feedback mechanism (Fig. 2G).

To assess the pathway-level consequences of the likely dual effects of the p.R158L pDM in TP53, we used iPAGE^12^, an information-theoretic pathway analysis framework, that calculates statistical significance of enriched pathway using a randomization-based statistical test. We determined gene expression changes in the lung cancer samples carrying p.R158L mutation relative to other samples in the same lung cancer cohorts^7^,^8^ and specified them as input for iPAGE. Interestingly, we found several known apoptosis and TP53-associated pathways were affected in the samples with the TP53 p.R158L mutation (Supplementary Fig. 5). To identify specific genes changed in these pathway, we integrated pathway-level data from the KEGG^13^ and identified the TP53 target genes involved in different biological pathway such as apoptosis, DNA repair and angiogenesis. We found that many of the TP53 target genes had systematic expression changes in the lung cancer samples carrying p.R158L mutation. Additionally, these changes in expression patterns were consistent at the pathway level as well. For example, TP53 transcriptionally activates CDKN1A (p21), which in turn suppresses CDK4 leading to G1 arrest and subsequently cause cell cycle arrest in normal cells, and p.R158L mutation is expected to affect the normal TP53 function. Consistently, we observed that the samples with p.R158L pDM had low CDKN1A expression relative to other samples in the cohort.

**Figure 3:**
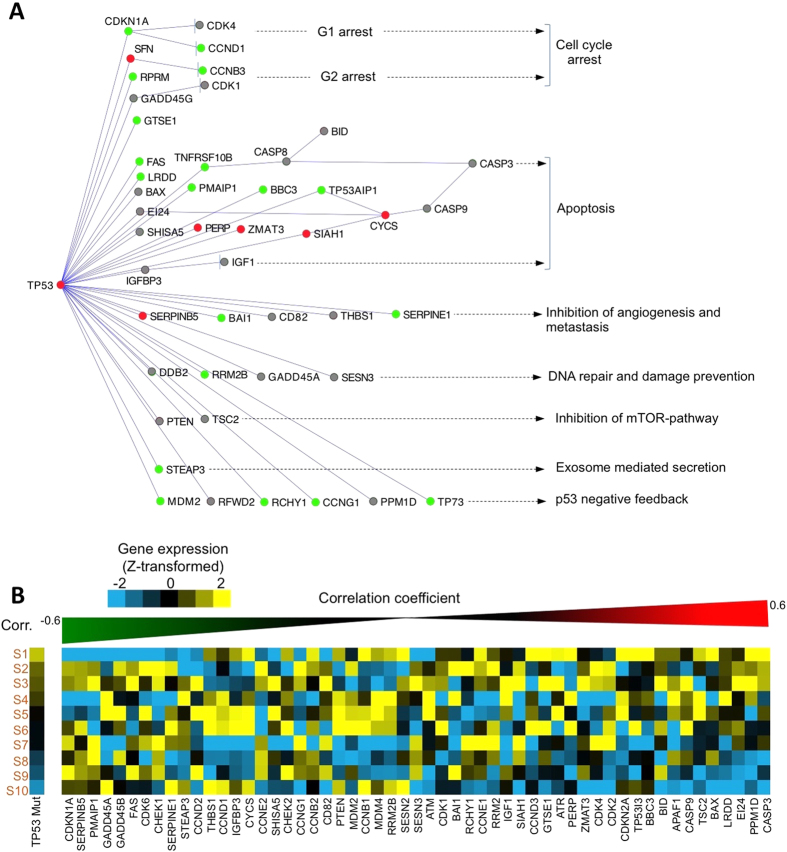
Pathway level changes associated with the potential duon mutation in TP53. (**A**) Direct targets of TP53 and downstream pathways are shown, with expression patterns in the lung cancer cohorts super-imposed. Expression patterns of TP53 and its direct and indirect downstream targets were compared between the lung cancer samples that have TP53 R158L mutation (cases) and others (controls). If expression of a gene was systematically higher, lower, or comparable in the cases relative to controls, those are shown in red, green, or grey respectively. (**B**) The lung cancer samples that have TP53 p.R158L mutation were ranked based on the allelic expression of the mutant gene copy, and indeed the direct and indirect downstream targets show consistent changes in expression. For instance, CDKN1A, which is the direct target of TP53, has reduced expression with an increase in the expression of the mutant copy of TP53.

One might ask whether the effects of the p.R158L mutation could be modulated by changes in expression of the mutant allele. To address this question, we focused on only the samples that have p.R158L mutations, and ranked them based on mutant TP53 allelic expression patterns. We observed systematic changes in the expression of the direct targets of TP53 (which can act as both activator and repressor), and also signature genes in the downstream pathways, especially cell cycle regulation and metastasis (Fig. 3A,B). Our results suggest that, the pDM accomplished three objectives in a single hit–it created a TP53 gene copy with abnormal function, was associated with increased expression of the abnormal gene-product, and also suppressed expression of the TP53 wild type gene copy via TP53 feedback loop–ultimately amplifying the functional consequences in the downstream pathways. We did not observe similar association between expression levels of TP53 and CDKN1A when samples with other TP53 mutations or wild type TP53 were analyzed (Supplementary Figs 6 and 7), indicating that expression variation of TP53 in the samples with wild type TP53 or other TP53 missense mutations did not have similar effects on the TP53 downstream targets such as CDKN1A.

### Missense pDM in SF3B1

**Figure 4:**
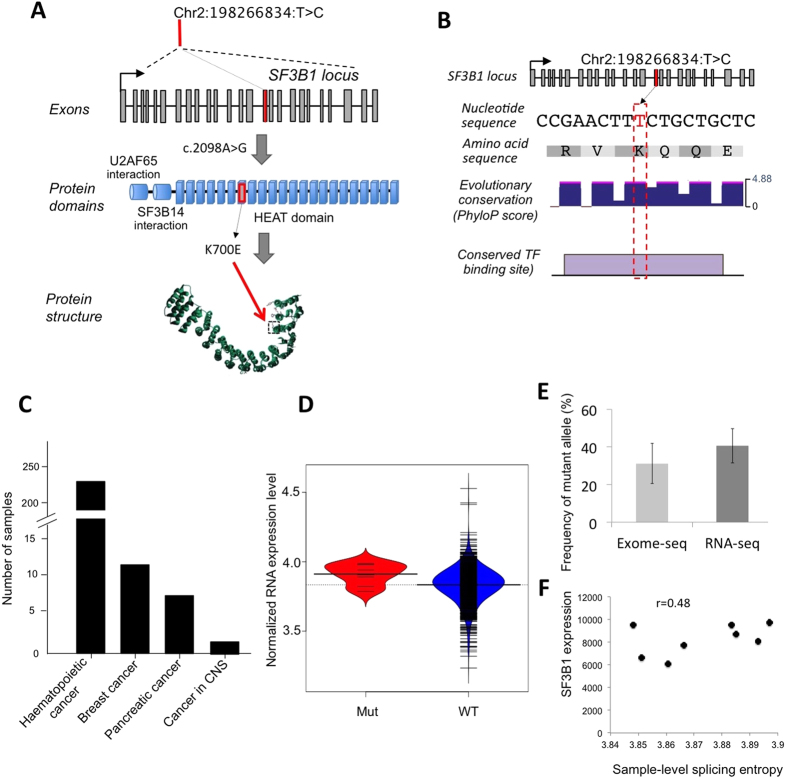
A somatic mutation with signatures of potential duon mutation in SF3B1. (**A**) Location of the pDM (Chr2:198266834:T > C) in the coding region of SF3B1 is shown. (**B**) Summary diagram showing base-by-base Phastcons evolutionary conservation and predicted transcription factor (HSF1) binding motif. (**C**) Presence of the pDM in in different cancer types. (**D**) Beanplot showing SF3B1 mRNA level expression of the samples carrying p.K700E pDM, compared to other samples that are wild type at that site in the breast cancer cohort. Statistical significance was estimated using Mann Whitney U test, *P*-value = 0.03. (**G**) Frequency of mutant allele at DNA and RNA levels determined by exome-seq and RNA-seq respectively in breast cancer samples. Plot represents mean allelic frequency for mutant allele with 95% CI of mean as error bars.

Another missense mutation in SF3B1 (Chr2:198266834:T > C; p.K700E), detected in eight breast cancer samples, also carried the signatures of pDM. The p.K700E mutation was present in the HEAT-repeat domain that is involved in mRNA splicing (Fig. 4A)^14^,^15^,^16^. The nucleotide position had high evolutionary conservation and overlapped regulatory motifs (Fig. 4B). This mutation was also reported in other cancer types including haematopoietic, pancreatic and central nervous system cancers^10^ (Fig. 4C). The samples with SF3B1 p.K700E mutation had relatively higher expression of the mutant allele, which in turn contributed to higher overall mRNA expression (Fig. 4D,E). Additionally, none of the affected samples had detectable copy number alteration.

To determine the dual effects of the mutation, we investigated if expression level of the mutant allele correlated with splicing abnormalities at the transcriptome-wide level. For each sample, we obtained isoform level mRNA expression data, and calculated sample-level splicing entropy using a method by Ritchie *et al.*^17^. We focused on the breast cancer samples that had p.K700E mutation in the cohort, ranking them based on SF3B1 mRNA expression level; we found positive correlation (r = 0.48) between the pDM expression and sample-level splicing entropy (Fig. 4F). The association was even stronger (r = 0.84) when we excluded a single apparent outlier. Again, we did not observe similar association between SF3B1 expression and splicing entropy when samples with wild type SF3B1 were investigated (Supplementary Fig. 8), indicating variation in expression of wild type copy of SF3B1 alone is unable to recapitulate the observed effects.

To evaluate pathway level significance of the SF3B1 p.K700E pDM, we estimated isoform abundance for each gene, and accordingly calculated gene-level splicing entropy. We then calculated the correlation coefficient between splicing entropy of gene and SF3B1 expression across the eight breast cancer samples, and use those correlation coefficients as input for the iPAGE. This analysis showed enrichment for VEGF pathway and matrix metalloproteinases, indicating that high expression of the p.K700E was associated with changes in splicing patterns in pathways, especially those involved in angiogenesis and matrix degradation (Supplementary Fig. 9).

### Other classes of pDM

In addition to missense pDMs, we also detected 30 in-frame indels that satisfied the criteria for pDMs, of which 27 were deletions and 3 were insertions. Interestingly, we found in-frame deletion pDM in two cancer genes WRN (c2305-2307; ACTAAAGAA > ACTAAA, pK506-E507 deletion, REDACT score: RED*cT) and CBL (c1506-1508; AATTATGAT > AATTAT, pY455-D456 deletion, REDACT score: REd*cT) in pancreatic adenocarcinoma (PAAD). The WRN pDM deletion was present in five PAAD samples; it was associated with expression changes and overlapped with DNase hypersensitive sites and the binding motif of PU.1. The CBL pDM deletion was detected in eight PAAD samples, it was associated with up-regulation of this gene, and overlapped SOX5 binding site. The number of in-frame insertion pDM was smaller. In particular, we detected an in-frame insertion in ERBB2 (chr17:37880981, LUAD; 5 samples) that mapped to a DNase hypersensitive site and was associated with considerable over-expression of ERBB2.

To identify pDMs with probable gain of transcription factor binding sites we scanned the exonic sequences with known position-weight matrix for transcription factors. We identified the cases where the exonic sequence carrying mutant copy had a predicted transcription factor-binding site, but the one with the wild-type allele had none (or binding site of a different factor). We found 4 pDMs that were predicted to generate new binding site for transcription factors; 3 of them were in cancer genes (Supplementary Table 4). As an example, we found a recurrent TP53 mutation (in breast and head neck cancer) that was predicted to result in gain of NHLH1 binding site, and was associated with down regulation of TP53 expression in both cancer type.

## Discussion

Integrating evidence from overall mRNA expression, allelic expression, regulatory motif perturbation, and chromatin signatures, we identify potential duon mutations in 4606 samples from 19 different cancer types. Our analysis suggests that somatic mutations with potentially duon functions might be common, and could affect cancer genes in multiple different malignancies. Our computational analysis indicates that such mutations can have complex functional consequences on downstream pathways, where altered expression can act as a modifier.

We note potential technical caveats. First, intra-tumor spatial heterogeneity has the potential to introduce discrepancies when comparing mRNA and protein expression levels, or allelic proportions in exome and RNAseq data from the same tumor sample. By looking for consistent signatures across different REDACT features we have probably minimized the false-positive cases, but the stringent filters could compromise on sensitivity. Second, despite recent advances in statistical and computational approaches, integrative analysis of large-scale diverse data types remains non-trivial. Third, statistical significance of overlapping regulatory features may not adequately reflect their biological importance, and overlap with regulatory features might be biologically relevant even when the associated statistical significance is weak. For instance, mutations in a gene with DNase hypersensitive sites spanning most of the coding regions cannot have significant p-value for D, but it still may be functionally relevant while classifying functional relevance of these mutations. Thus, we conservatively interpret the combined p-values in conjunction with the REDACT score. Fourth, we did not consider the potential duon mutations, which could alter the expression of the gene products by post-transcriptional or post-translational modifications. Finally, detailed functional characterization of the pDMs was beyond the scope of this paper. Even though the pDMs had multiple lines of support, we recommend caution while inferring causality and mechanism of regulation from correlation, especially where experimental evidence was limited.

Nevertheless, our findings present evidence for recurrent, potential duon mutations in the genomes of different types of cancer. Our results challenges the established paradigm of assessing significance of the mutations in protein-coding genes primarily on protein structures, and calls for an integrative approach to assess additional consequences of these mutations. Furthermore, our study suggests that potential duon mutations in cancer genes may have under-appreciated significance for downstream pathways. It can be possible to extend our analysis to cases of different cancer types, and also in other diseases where duon mutations are common. Lastly, our findings highlight the impact of regulatory mutations in tumorigenesis and contribute to the ongoing debate about the early molecular alterations during tumor development^18^.

## Methods

### Data acquisition

We obtained the catalog of somatic, exonic mutations (e.g. point mutations, small InDels) for 4606 samples from 19 different cancer-types, as provided by the Cancer Genome Atlas (TCGA; https://tcga-data.nci.nih.gov/tcga/). There were a total of 1,061,980 mutations reported (see Supplementary Table 1 for the summary). We also obtained RNA and protein-level expression, tumor purity and exonic allele frequency data, as well as other clinical data-types from the TCGA. Pre-computed gene-level copy number alteration status for selected loci were obtained from the MSKCC cbio portal.

We integrated data from the dbSNP and excluded all TCGA somatic mutations that overlapped with the catalog of common genetic variants (e.g. SNPs) in the population. Genomic regions with potential regulatory function were obtained from the RegulomeDB^6^, which integrates published transcription factor binding site motif and chromatin immunoprecipitation data, together with DNase hypersensitivity and motif-level evolutionary conservation data to infer regulatory activity of a region. Additionally, to infer the effect (loss- or gain-of transcription factor binding site) of the somatic mutations we integrated position-weight matrix data for sequence-specific transcription factors using Jasper^19^. Base-by-base evolutionary conservation data using GERP++ and reference functional datasets from the ENCODE project were obtained from the UCSC Genome Browser. The catalog of cancer genes was obtained from the COSMIC^10^.

### Potential duon mutation and REDACT score

We developed the REDACT scoring system to identify recurrent pDMs and summarize their supporting evidence: (i) ***R***ecurrence (at least 3 samples or >1% samples, whichever is greater), (ii) mRNA-level ***E***xpression change (iii) overlap with ***D***Nase hypersensitive regions, (iv) ***A***llelic expression imbalance, (v) overlap with transcription factor ***C***hIP-seq or ChIP-chip peaks, and (vi) overlap with predicted ***T***ranscription factor binding motifs. In the REDACT score, each letter indicates the status of the corresponding supporting evidence. If the supporting data-type is present but the evidence is not consistent, we present it in lowercase; on the other hand, if the supporting data type is absent we present it with an asterisk. For instance, if a pDM score of ‘REd*Ct’ would indicate that the mutation is recurrent, associated with altered mRNA expression and ChIP-seq peaks, but it does not overlap with DNase hypersensitive regions and known transcription factor binding motifs, while allelic expression data is not available. Since protein-level expression data is available for only a small number of gene-products, we report it on a case-by-case basis. All mutations reported in this study are recurrent (R), associated with altered mRNA expression (E), and have at least one type of regulatory evidence (D, C, or T) available in support.

Statistical significance at the level of *E* was estimated by comparing RNA expression in the affected samples to that in the other samples in the cohort using Mann Whitney U test. P value at the level of *A* was computed using binomial test on the allelic proportions in the RNAseq data, with allelic proportions in the corresponding exome data as prior. To calculate p-values for regulatory evidence (D, C, and T), we performed permutation analysis. We note that permutation could be performed using a number of different strategies (e.g. controlling for nucleotide or chromatin context during shuffling); but in some cases such strategies constrained the search space and increased the risk of over-sampling. So, to estimate statistical significance of overlap with DNase hypersensitive sites, we used a simple null model, and randomly shuffled the somatic mutations within respective candidate gene regions 10,000 times, and counted the number of times (*n*) the mutations overlap with DNase hypersensitive regions, by chance alone, such that n/10000 indicates the permutation p-value. Similarly, we calculated p-values for overlap with TFBS motifs, and ChIP peaks. False discovery rate (FDR) was used for multiple testing corrections.

We combined the unadjusted p-values using Fisher’s method, assuming the lines of evidence are independent. We also used an alternative strategy using Hartung’s method^20^,^21^, which enable combining correlated p-values; the key conclusions remained consistent (Supplementary Table 2). Nevertheless, we recognize challenges in computing, combining, and interpreting signals from heterogeneous data types^20^,^22^, and consider pDMs based on both the combined p-value and the REDACT score.

### Functional analysis

To ascertain functional consequences of the mutations we integrated both structural and pathway-level data. For instance for the analysis of TP53 pDM, we obtained list of TP53 target genes involved in different biological pathway such as apoptosis, DNA repair and angiogenesis^23^,^24^. Furthermore, wherever possible, we superimposed the known regulatory relationship among these genes. For example, TP53 transcriptionally activate CDKN1A (p21) and p21 suppresses CDK4 that regulate G1 arrest and subsequently cause cell cycle arrest in normal cells. In our functional analysis we considered the direction of change in expression. Additionally, two-control analyses were performed to show effects of both p.R158L missense mutation and associated expression changes. First, to test whether any missense mutation in TP53 systematically affect CDKN1A expression in a fashion similar to p.R158L, we selected several other TP53 somatic mutations that were recurrent in different TCGA cancer types (Lower grade glioma (LGG), Lung squamous cell carcinoma (LUSC), Lung adenocarcinoma (LUAD), Head and neck (HNSC), and Bladder (BLCA) cancers), but were not classified as duon mutations; we estimated correlation in RNAseq expression between TP53 and CDKN1A (Supplementary Fig. 6). Second, to test whether the extent of association between TP53 and CDKN1A expression for the TP53 p.R158L mutant samples is rather common among TP53 wild type samples, we performed permutation analysis. We randomly selected 10 samples wild-type TP53, 1000 times from the LUAD and LUSC cohorts of TCGA, and each time calculated correlation coefficient value between expression of CDKN1A and TP53, and then compared that with the observed association between TP53 and CDKN1A expression in p.R158L mutant samples (Supplementary Fig. 7).

For functional analysis of SF3B1 mutation, as SF3B1 is a core spliceosome factor our aim is to evaluate the extent of non-specific alternative transcript disruptions in the samples containing mutation in SF3B1. We hypothesized that, in a samples where SF3B1 is mutated the splicing machinery is impaired, the distribution of isoforms may be more disordered than in unaffected samples and to quantify this we modified previously published method of calculating isoform entropy by Ritchie *et. al*.^17^ Isoform entropy is calculated using Shannon’s entropy index. In brief, if a random variable *X* has values *x*_*i*_ (*x*_*i;*_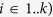 with probabilities *P* (*x*_*i*_) such that *P* (*x*_*i*_) ≥ 0 and 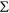 P (*x*_*i*_) = 1 then Shannon’s entropy index is defined by:


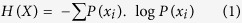


Here we calculated gene by gene splicing entropy after considering relative abundance of all expressed isoforms a genes in that sample. We also calculated sample level splicing entropy after considering relative abundance of all expressed isoforms across all genes in that sample.

For pathway analysis we used iPAGE, a mutual information-based approach to discover the enriched pathways in samples with a pDM of interest. For the TP53 pDM analysis, we transformed the expression data using the equation:


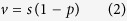


where for any gene p is the Student’s t-test p-value between the mutant samples and other samples in the same lung cancer cohorts, and s indicates the direction of change in the expression between the two groups of samples. Thus, v indicates the extent to which a gene is up-regulated or down-regulated in the mutant samples relative to other samples in the cohort with maximal and minimal values of 1 and −1 respectively. For the pDM in SF3B1, we calculated entropy for each gene in eight samples of breast cancer that contains p.K700E mutation. Next, we calculated correlation coefficient between entropy of gene and SF3B1 expression across eight samples and used that value as input for iPAGE analysis. Similar to the control analysis for TP53, randomly selected 8 samples wild-type SF3B1, 1000 times from the TCGA BRCA cohort, and each time calculated correlation coefficient value between expression of SF3B1 and splicing entropy, and compared that with the observed association between SF3B1 expression and splicing entropy in p.K700E mutant samples (Supplementary Fig. 8).

## Additional Information

**How to cite this article**: Yadav, V. K. *et al.* Significance of duon mutations in cancer genomes. *Sci. Rep.* **6**, 27437; doi: 10.1038/srep27437 (2016).

## Supplementary Material

Supplementary Information
