# Supplementary material for: Significance of duon mutations in cancer genomes
Source: Sci Rep. 2016 Jun 8;6:27437. doi: 10.1038/srep27437 (PMC4897603; doi:10.1038/srep27437)
Supplement: Supplementary Information [file srep27437-s1.pdf]

Supplementary Information

for

Significance of duon mutations in cancer genomes

Vinod Kumar Yadav[1,2*], Kyle S. Smith[1, 3], Colin Flinders[4], Shannon M. Mumenthaler[4], Subhajyoti De[1, 5, 6*]

[1] Department of Medicine, University of Colorado School of Medicine, Aurora, CO 80045, USA; [2] The Jackson Laboratory, Farmington, CT06032, USA; [3] Computational Biosciences Graduate Program, University of Colorado, Aurora, CO 80045, USA [4] Center for Applied Molecular Medicine, University of Southern California, Los Angeles, CA, 90033, USA; [5] University of Colorado Cancer Center, Aurora 80045, CO, USA; [6] Rutgers Cancer Institute of New Jersey, New Brunswick, NJ 08817; *Email: vinodyadav2083@gmail.com, subhajyoti.de@rutgers.edu

Analysis pipeline used to identify cancer specific Duon Mutations

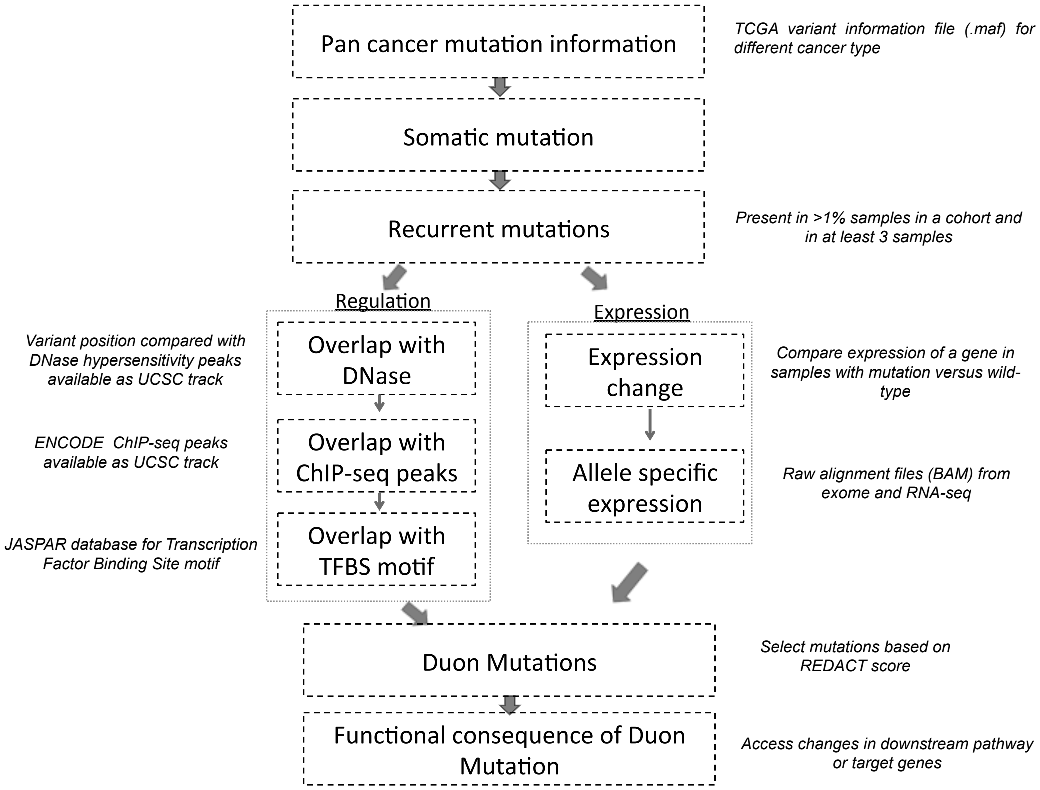

Supplementary Figure S1: Analysis pipeline used to detect duon mutations in different cancer type. Our dataset had a total of 1,061,980 mutations, which were mapped to 930,697 unique genomic locations. Of them 1,594 were recurrent by our criteria. 152 of the recurrent mutations had significant expression changes. Allelic expression data was calculated for a minor subset. Assessment of regulatory features was done in parallel. 711, 712, and 131 recurrent mutations overlapped with DNase hypersensitive regions, Chipseq peaks, and predicted transcription factor binding motifs, respectively. Silent, nonsense, frame-shift, and splice site mutations were excluded. The final catalog had 50 potential duon mutations with REDACT score.

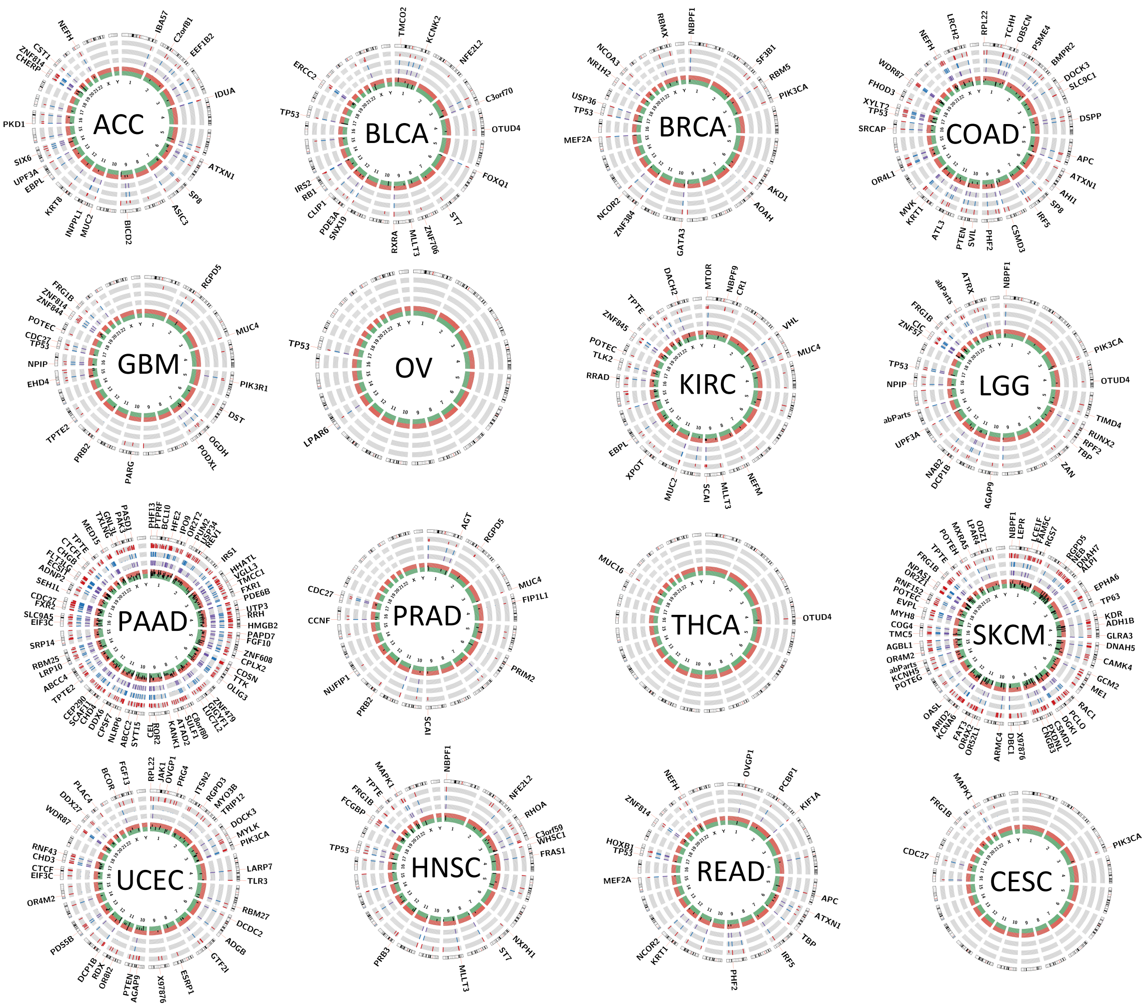

Supplementary Figure S2: Circos plot showing the genome-wide landscapes of recurrent coding mutations, including those that overlap with DNase hypersensitive cluster, transcription factor ChIP-seq peaks, and altered expression of the genes that harbor them, for different cancer type. Only DNase regions, ChIP-seq peaks overlap with recurrent mutations were shown in the plot.

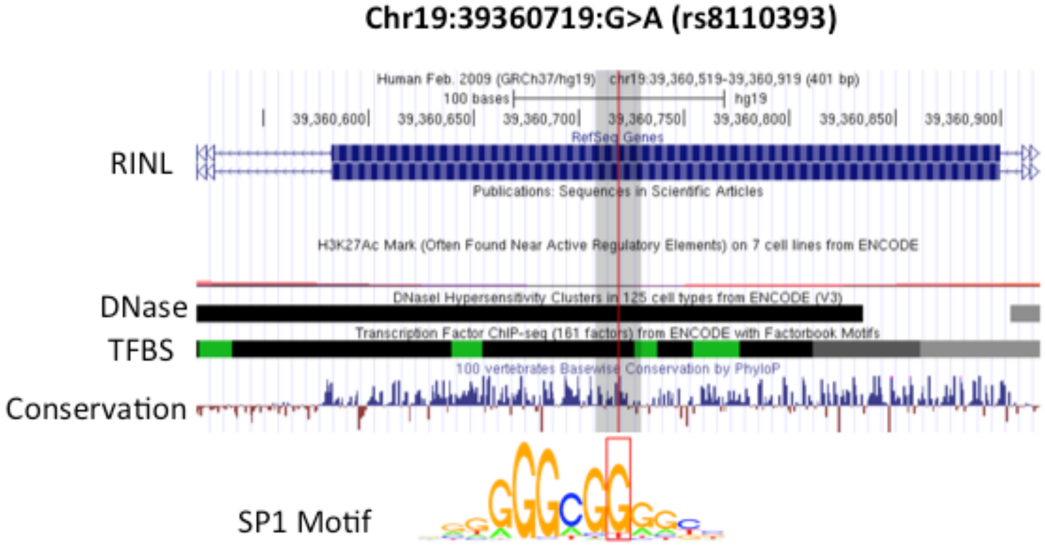

Supplementary Figure S3: To show utility of the REDACT scoring system for a "normal dataset". Supporting evidence for SNP rs8110393. It is recurrent with population allele frequency > 1% in the 1000 Genomes Project cohort. It is a known eQTL variant, i.e. there is significant association between rs8110393 and allelic expression of ECH1, a proximal gene (http://eqtl.rc.fas.harvard.edu/eqtlbrowser/metamrcaelist/10173. It overlaps with DNase hypersensitive region, ChIP peaks and TFBS motif.

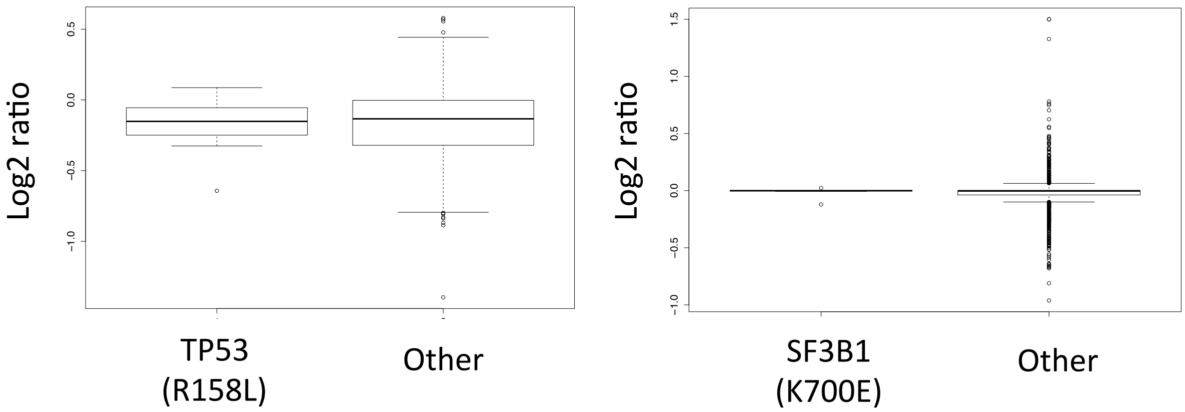

Supplementary Figure S4: Boxplot for (Left panel) TP53 copy number signal intensity log2 ratio based on Affy SNP6 arrays for the lung cancer samples containing TP53 p.R158L mutation and other samples from the TCGA LUAD and LUSC cohorts, and (Right panel) SF3B1 copy number signal intensity log2 ratio based on Affy SNP6 arrays for the breast cancer samples containing SF3B1 p.K700E and other BRCA samples from the TCGA (Right panel). Samples with TP53 p.R158L mutation and SF3B1 p.K700E mutation had no systematic bias for copy number deletion compared to other samples in respective cohorts (Mann Whitney U test; p value > 0.05 for both cases).

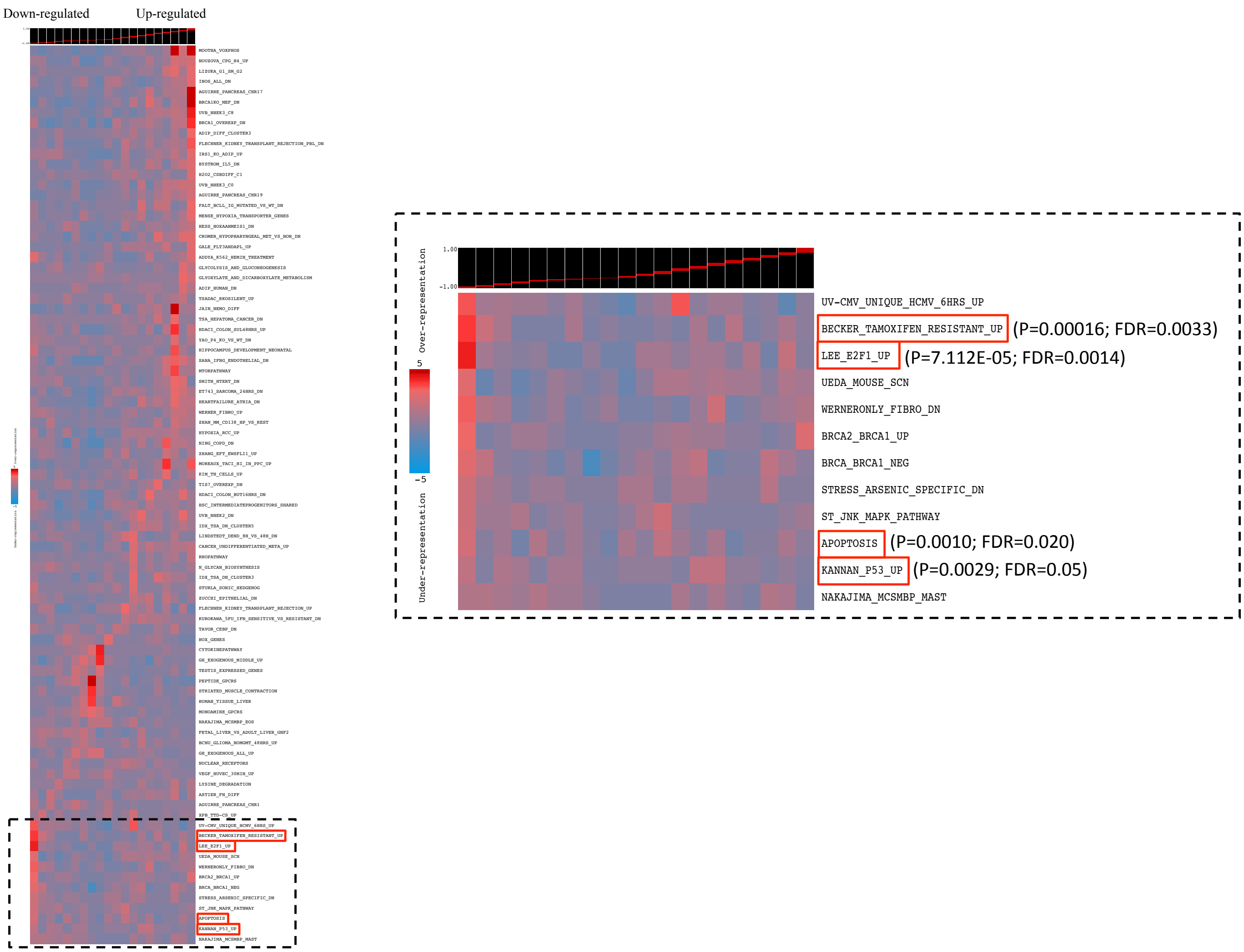

Supplementary Figure S5: Shown are the gene-sets discovered by iPAGE and their patterns of overrepresentation across the R158L (TP53) containing lung cancer samples versus rest of samples of TCGA lung cancer expression difference. These differences are partitioned into discrete expression bins. Each expression bin includes genes within a specific range of expression values (shown in the top panel). Bins to the left contain genes with lower ex FETAL_LIVER_VS_ADULT_LIVER_GNF2 in mutant samples, whereas the ones to the right contain genes with higher expression. In the heat map representation, rows correspond to pathways and columns to consecutive expression bins. Red entries indicate enrichment of gene-sets genes in a given expression bin. Enrichment and depletion are measured using hyper geometric p values (log-transformed). In insert gene-set overrepresented in down-regulated genes were shown. Overrepresented in down-regulated genes were shown.

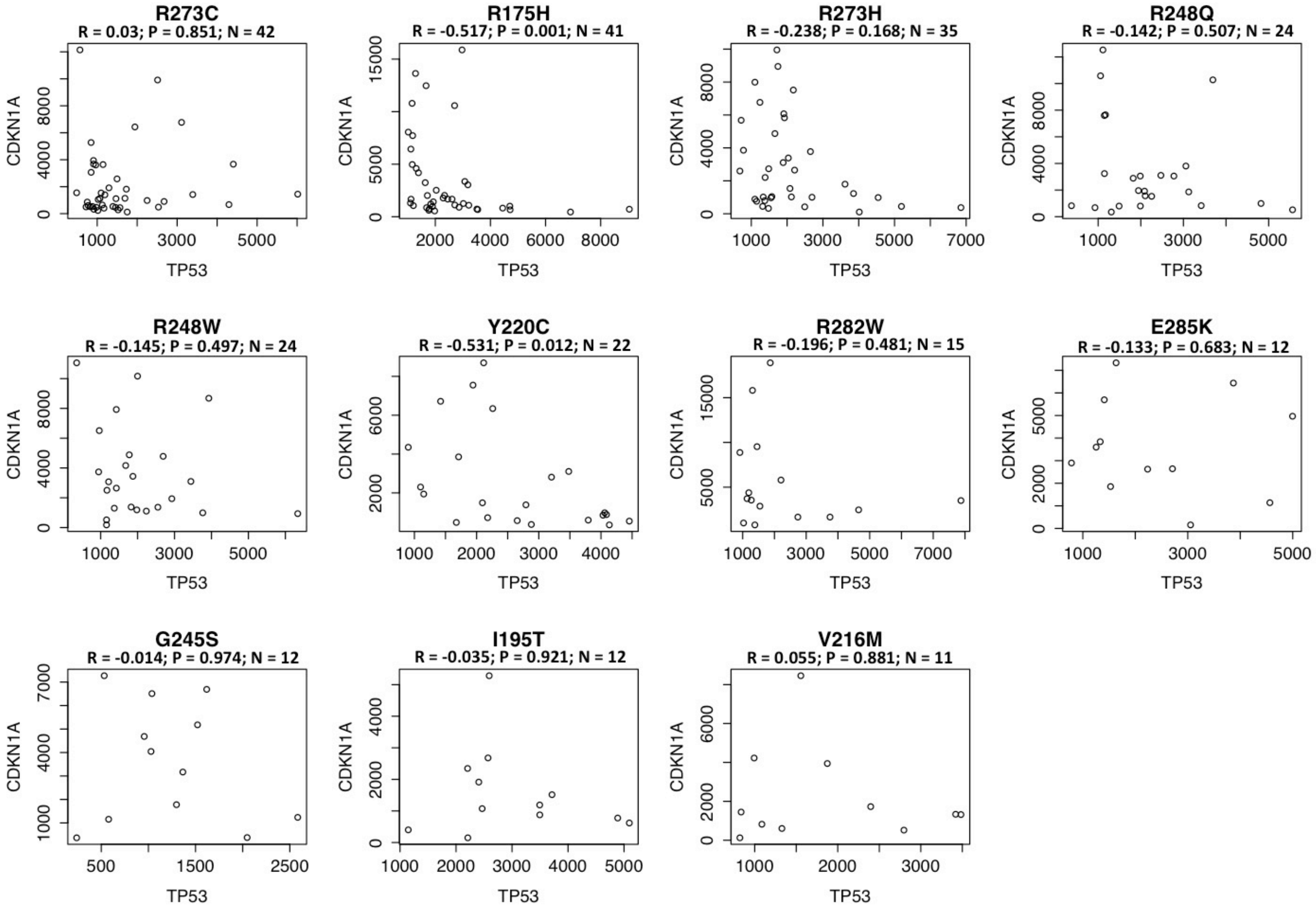

Supplementary Figure S6: To test whether any missense mutation in TP53 systematically affect CDKN1A expression in a fashion similar to R158L, we selected several other TP53 somatic mutations that were recurrent in one or more TCGA cancer types, but were not classified as duon mutations. Plotting TP53 and CDKN1A expression for these cases using RNAseq data from the TCGA cancer cohorts, we found no universal patterns.

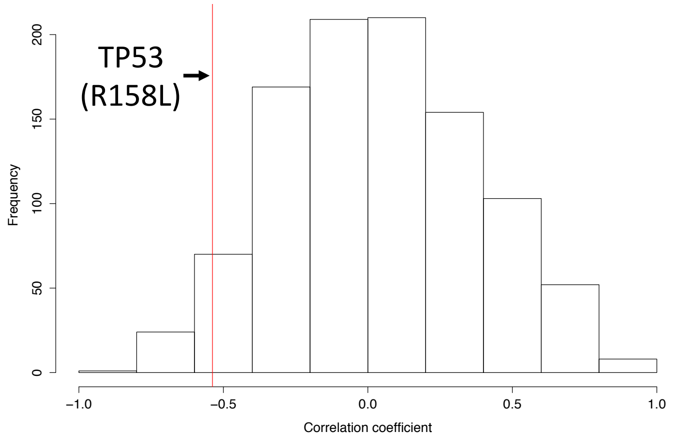

Supplementary Figure S7: To test whether the extent of association between TP53 and CDKN1A expression for the TP53 p.R158L mutant samples is rather common among TP53 wild type samples, we performed permutation analysis. We randomly selected 10 samples wild-type TP53, 1000 times from the LUAD and LUSC cohorts of TCGA, and each time calculated correlation coefficient value between expression of CDKN1A and TP53. The distribution of correlation value is shown; the red line represents correlation coefficient for TP53 and CDKN1A expression in samples containing TP53 R158L mutation (Pearson correlation coefficient: -0.54; permutation p-value: 0.045).

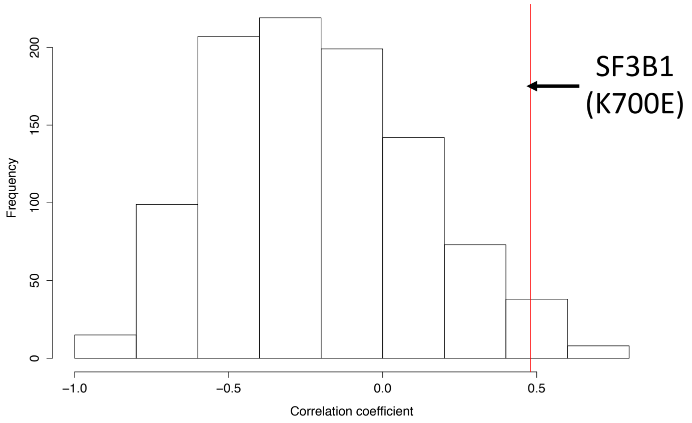

Supplementary Figure S8: To test whether the extent of association between SF3B1 expression and splicing entropy for the SF3B1 p.K700E mutant samples is rather common among SF3B1 wild type samples, we performed permutation analysis. We randomly selected 8 samples wild-type SF3B1, 1000 times from the TCGA BRCA cohort, and each time calculated correlation coefficient value between expression of SF3B1 and splicing entropy. The distribution of correlation value is shown; the red line represents correlation coefficient for SF3B1 expression and splicing entropy in the samples containing SF3B1 p.K700E mutation (correlation coefficient: 0.48; permutation p-value: 0.027)

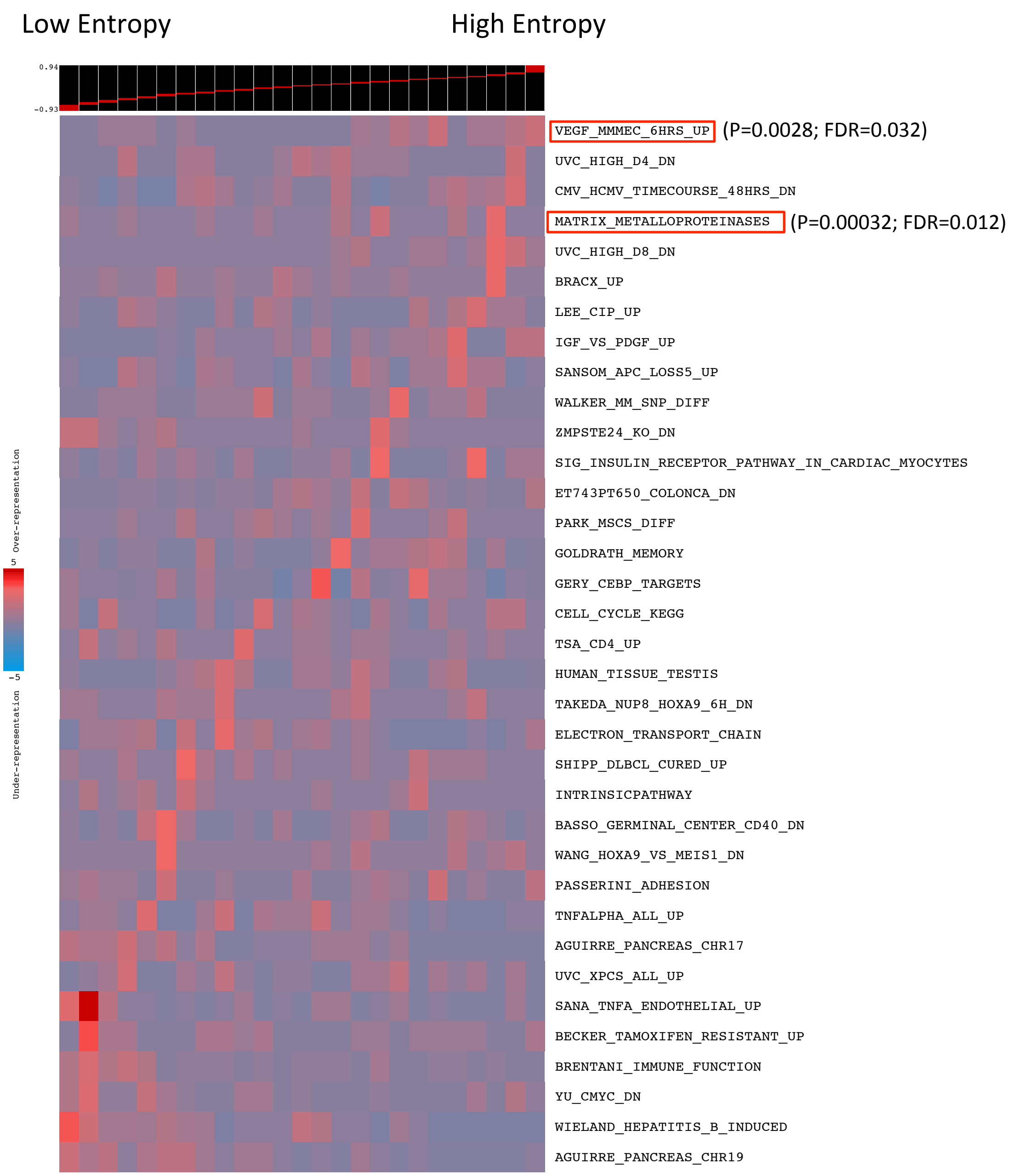

Supplementary Figure S9: Shown are the gene-sets discovered by iPAGE and their patterns of overrepresentation across the K700E (SF3B1) containing breast cancer samples versus rest of samples of TCGA breast cancer entropy difference. These differences are partitioned into discrete bins. Each bin includes genes within a specific range of entropy values (shown in the top panel). Bins to the left contain genes with lower entropy in mutant samples, whereas the ones to the right contain genes with higher entropy. In the heat map representation, rows correspond to gene sets and columns to consecutive entropy bins. Red entries indicate enrichment of gene-sets genes in a given bin. Enrichment and depletion are measured using hypergeometric p values (log-transformed).

Supplementary Table 1:- Number of samples, somatic and recurrent mutations found in pan-cancer analyses

Cancer Type	Sample Numbers	Somatic Mutations	Recurrent Mutations
ACC	91	13130	57
BLCA	130	7558	35
BRCA	776	47244	21
CESC	39	10021	7
COAD	219	114470	97
GBM	291	22167	51
HNSC	306	74009	71
KIRC	297	26266	74
LAML	197	2586	10
LGG	220	27316	61
LUAD	538	234279	319
LUSC	178	65306	19
OV	142	6309	2
PAAD	57	27746	357
PRAD	83	5918	25
READ	102	25004	34
SKCM	341	289506	264
THCA	405	7459	3
UCEC	194	55686	88
**Total**	**4606**	**1061980**	**1595**

Supplementary Table 2: The catalog of somatic potential duon mutations in the cancer cohorts.

Cancer type	genename	chr	start	end	Ref	Alt	mutation	Type	Sample number mutation present	Cancer associated genes (Y or N)	Score (REDACT)	FDR corrected P-value Expression	FDR corrected P-value Dnase	FDR corrected P-value ChIP-seq	FDR corrected P-value TFBS	FDR corrected P-value Allelic expression	fishers fdr adjusted	hartung fdr adjusted
READ	PCBP1	2	70315174	70315174	T	A	Missense_Mutation	SNP	3	N	REDaCt	0.01616467	2.50E-11	2.50E-11	NA	NA	1.00E-16	2.05E-30
SKCM	KIAA0907	1	155887393	155887393	T	G	Missense_Mutation	SNP	5	N	REdacT	0.03430667	NA	NA	0.00025	5.00E-16	1.00E-16	2.50E-24
ACC	BICD2	9	95526977	95526977	G	T	Missense_Mutation	SNP	3	N	REDaCt	0.0429225	1.67E-04	1.67E-04	NA	1.47E-08	1.05E-13	9.10E-20
KIRC	MUC4	3	195505836	195505836	G	C	Missense_Mutation	SNP	5	N	REdacT	0.05641667	NA	NA	0.00025	1.72E-10	5.88E-13	1.49E-18
LUSC	TP53	17	7578457	7578457	C	A	Missense_Mutation	SNP	5	Y	REDACT	0.03194333	1.18E-01	1.74E-01	0.00025	5.00E-16	1.00E-16	2.60E-17
LUAD	TP53	17	7578457	7578457	C	A	Missense_Mutation	SNP	5	Y	REDACT	0.0517	1.19E-01	1.75E-01	0.00025	5.00E-16	1.00E-16	5.94E-17
COAD	KCNN3	1	154842244	154842244	A	T	Missense_Mutation	SNP	7	N	REDAct	0.115735	2.65E-01	NA	NA	5.00E-16	7.59E-15	1.60E-15
HNSC	RANGAP1	22	41652800	41652800	A	C	Missense_Mutation	SNP	4	N	REDACT	0.0254275	1.67E-02	1.48E-01	0.01666667	8.30E-13	8.23E-14	4.04E-15
HNSC	NFE2L2	2	178098810	178098810	C	G	Missense_Mutation	SNP	5	Y	REdAcT	0.032457	NA	NA	0.00025	4.14E-06	3.64E-09	9.10E-15
SKCM	RGPD5	2	113127775	113127775	G	C	Missense_Mutation	SNP	5	N	REdacT	0.06432333	NA	NA	0.00025	5.60E-05	6.94E-08	1.07E-12
SKCM	TEX15	8	30705003	30705003	C	T	Missense_Mutation	SNP	6	N	REd*CT	0.07958	NA	2.50E-04	0.00025	NA	5.29E-07	3.31E-09
ACC	CHERP	19	16640581	16640583	TGC	-	In_Frame_Del	DEL	3	N	RED*cT	0.0124385	1.73E-01	NA	0.0005	NA	2.92E-05	3.78E-08
PAAD	WRN	8	30945377	30945379	AAG	-	In_Frame_Del	DEL	5	Y	RED*cT	0.06649333	2.50E-04	NA	0.00025	NA	4.87E-07	8.18E-08
PAAD	RABEP2	16	28931200	28931202	CTG	-	In_Frame_Del	DEL	5	N	RED*cT	0.0137655	3.69E-01	NA	0.0005	NA	5.87E-05	2.94E-07
BRCA	NBPF1	1	16902844	16902844	G	T	Missense_Mutation	SNP	12	N	RED*cT	0.074275	2.64E-01	NA	0.0005	NA	1.49E-04	1.45E-06
KIRC	EBPL	13	50235160	50235160	G	C	Missense_Mutation	SNP	5	N	REDAct	0.0159815	3.73E-01	NA	NA	3.32E-03	2.61E-04	2.35E-06
PAAD	FUZ	19	50310483	50310485	CAG	-	In_Frame_Del	DEL	11	N	RED*CT	0.0007335	5.61E-01	5.61E-01	0.0005	NA	2.58E-05	3.87E-06
LGG	CIC	19	42791757	42791757	C	T	Missense_Mutation	SNP	5	Y	REDAcT	0.009077	7.41E-01	NA	0.23183333	3.56E-04	6.98E-05	7.95E-06
BRCA	GIGYF2	2	233712227	233712229	ACA	-	In_Frame_Del	DEL	11	N	REd*cT	0.06077	NA	NA	0.0005	NA	9.19E-05	3.15E-05
SKCM	SIGLEC6	19	52034594	52034594	C	T	Missense_Mutation	SNP	6	N	REd*Ct	0.046655	NA	5.00E-04	NA	NA	7.55E-05	7.30E-05
PAAD	ASXL2	2	26022304	26022306	CTG	-	In_Frame_Del	DEL	8	N	REd*cT	0.0391565	NA	NA	0.0005	NA	6.98E-05	1.06E-04
BRCA	SF3B1	2	198266834	198266834	T	C	Missense_Mutation	SNP	8	Y	REdAcT	0.05910667	NA	NA	0.0005	1.22E-02	1.26E-05	1.69E-04
PAAD	PLEKHG5	1	6536011	6536013	CTC	-	In_Frame_Del	DEL	7	N	RED*ct	0.013875	4.36E-01	NA	NA	NA	7.22E-03	3.51E-03
PAAD	GRM1	6	146755630	146755632	GAC	-	In_Frame_Del	DEL	4	N	RED*cT	0.019	2.78E-01	NA	0.019	NA	1.19E-03	9.25E-03
PAAD	LRP1	12	57605740	57605742	TGC	-	In_Frame_Del	DEL	4	N	RED*cT	0.016	2.67E-01	NA	0.016	NA	1.16E-03	9.25E-03
PAAD	CBL	11	119149356	119149358	ATG	-	In_Frame_Del	DEL	7	Y	REd*cT	0.023863	NA	NA	0.0255	NA	1.19E-03	1.32E-02
BRCA	NCOA3	20	46279837	46279839	CAG	-	In_Frame_Del	DEL	15	N	REd*cT	0.024948	NA	NA	0.12725	NA	4.58E-03	2.05E-02
PAAD	DAB2IP	9	124522389	124522391	AAG	-	In_Frame_Del	DEL	5	N	RED*cT	0.034283	2.09E-01	NA	0.03775	NA	2.07E-03	2.34E-02
PAAD	CCNT1	12	49087434	49087436	ATG	-	In_Frame_Del	DEL	4	N	REd*Ct	0.049024	NA	4.90E-02	NA	NA	4.58E-03	2.72E-02
COAD	PHF2	9	96438998	96438999	-	ACCACCCCTGCCTCC	In_Frame_Ins	INS	7	N	RED*ct	0.035842	3.00E-01	NA	NA	NA	1.10E-02	2.72E-02
PAAD	FADS2	11	61607885	61607887	TCC	-	In_Frame_Del	DEL	5	N	REd*cT	0.08738	NA	NA	0.069	NA	7.22E-03	3.44E-02
COAD	NCOR2	12	124824721	124824722	-	GCCGCTGCT	In_Frame_Ins	INS	9	N	RED*cT	0.09809	2.38E-01	NA	0.0495	NA	6.00E-03	4.19E-02
BRCA	TP53	17	7578271	7578271	T	C	Missense_Mutation	SNP	9	Y	RED*ct	0.065319	2.37E-01	NA	NA	NA	1.43E-02	4.27E-02
PAAD	TAS1R1	1	6638843	6638845	GCT	-	In_Frame_Del	DEL	4	N	RED*cT	0.07319	3.96E-01	NA	0.07319	NA	9.48E-03	4.30E-02
PAAD	RSBN1L	7	77326220	77326222	CCG	-	In_Frame_Del	DEL	4	N	RED*CT	0.123385	1.79E-01	1.79E-01	0.0305	NA	3.93E-03	4.44E-02
PAAD	EXTL1	1	26349533	26349535	CCT	-	In_Frame_Del	DEL	5	N	RED*ct	0.12706	1.92E-01	NA	NA	NA	1.96E-02	5.51E-02
PAAD	GIGYF1	7	100284335	100284337	CCT	-	In_Frame_Del	DEL	6	N	RED*CT	0.11516	2.45E-01	2.96E-01	0.054	NA	8.47E-03	5.51E-02
PAAD	MED15	22	20918916	20918918	CAG	-	In_Frame_Del	DEL	4	N	RED*CT	0.12451	3.79E-01	3.80E-01	0.0485	NA	1.27E-02	5.51E-02
PAAD	QRICH1	3	49094314	49094316	TGC	-	In_Frame_Del	DEL	7	N	REd*Ct	0.09268	NA	3.93E-01	NA	NA	2.52E-02	5.51E-02
PAAD	BPTF	17	65822267	65822269	GAG	-	In_Frame_Del	DEL	4	N	RED*Ct	0.059681	3.01E-01	3.01E-01	NA	NA	1.73E-02	5.82E-02
LUAD	ERBB2	17	37880981	37880982	-	GCATACGTGATG	In_Frame_Ins	INS	5	Y	RED*Ct	0.096208	2.02E-01	5.23E-01	NA	NA	2.44E-02	6.48E-02
PAAD	VAMP3	1	7838212	7838214	TCA	-	In_Frame_Del	DEL	8	N	REd*cT	0.17987	NA	NA	0.2115	NA	2.56E-02	6.48E-02
PAAD	ATP13A3	3	194181471	194181473	GAG	-	In_Frame_Del	DEL	4	N	RED*Ct	0.1211	2.27E-01	2.27E-01	NA	NA	1.89E-02	7.24E-02
HNSC	TP53	17	7578271	7578271	T	A	Missense_Mutation	SNP	5	Y	RED*ct	0.23475	2.35E-01	NA	NA	NA	3.55E-02	7.81E-02
PAAD	ROR2	9	94486026	94486028	TCC	-	In_Frame_Del	DEL	5	N	RED*ct	0.15271	6.68E-01	NA	NA	NA	5.40E-02	8.01E-02
LUSC	RNF213	17	78282825	78282825	G	A	Missense_Mutation	SNP	3	N	RED*Ct	0.21033333	2.10E-01	2.10E-01	NA	NA	2.46E-02	8.55E-02
LUSC	TP53	17	7578394	7578394	T	C	Missense_Mutation	SNP	3	Y	RED*Ct	0.2345	2.35E-01	2.91E-01	NA	NA	3.31E-02	9.67E-02
PAAD	FLT3LG	19	49978960	49978962	CTG	-	In_Frame_Del	DEL	6	N	RED*cT	0.23032	1.00E+00	NA	0.333	NA	9.27E-02	1.10E-01
PAAD	GRIN1	9	140056884	140056886	GAG	-	In_Frame_Del	DEL	4	N	RED*ct	0.22578	8.59E-01	NA	NA	NA	8.96E-02	1.10E-01
PAAD	TNFRSF9	1	7980912	7980914	CTT	-	In_Frame_Del	DEL	4	N	RED*CT	0.18159	4.29E-01	4.68E-01	0.2075	NA	4.47E-02	1.12E-01

Supplementary Table 3:- Clinical information of samples containing TP53 pDM in LUSC and LUAD

	Lung Squamous Cell Carcinoma (LUSC)				Lung Adenocarcinoma (LUAD)			
	All		TP53 Mutation (R158L)		All		TP53 Mutation (R158L)	
	Number	Percentage	Number	Percentage	Number	Percentage	Number	Percentage
	177		5	2.8	405		5	1.2
Sex								
Male	130	73.4	5	100.0	186	45.9	4	80.0
Female	47	26.6	0	0.0	219	54.1	1	20.0
Age								
<68	83	46.9	0	0.0	205	50.6	2	40.0
>=68	91	51.4	3	60.0	181	44.7	3	60.0
Missing	3	1.7	2	40.0	19	4.7	0	0.0
Tumor stage (T)								
T1	115	65.0	1	20.0	133	32.8	1	20.0
T2	36	20.3	3	60.0	224	55.3	1	20.0
T3+	26	14.7	1	20.0	48	11.9	3	60.0
Lymph node (N)								
N0	116	65.5	3	60.0	262	64.7	3	60.0
N1	39	22.0	2	40.0	75	18.5	1	20.0
N2+	22	12.4	0	0.0	67	16.5	1	20.0
Missing	0	0.0	0	0.0	1	0.2	0	0.0
Metastasis (M)								
M0	164	92.7	5	100.0	269	66.4	2	40.0
M1	3	1.7	0	0.0	18	4.4	0	0.0
MX	6	3.4	0	0.0	114	28.1	3	60.0
Missing	4	2.3	0	0.0	4	1.0	0	0.0
Death								
Dead	71	40.1	2	40.0	102	25.2	2	40.0
Alive/censored	106	59.9	3	60.0	303	74.8	3	60.0
Smoking								
<45	68	38.4	1	20.0	170	42.0	3	60.0
>=45	78	44.1	4	80.0	106	26.2	1	20.0
Missing	31	17.5	0	0.0	129	31.9	1	20.0

Supplementary Table 4:-List of pDMs identified with probable gain of function

Cancer Type	Genename	Chr	Start	End	Factor Name	mutation	Type	Ref	Alt	p-value expression	Expression Change (Mut vs. Wild-type)	Cancer associated genes (Y or N)
COAD	KCNN3	1	154842244	154842244	Gain of Myf binding site	Missense_Mutation	SNP	A	T	<0.05	UP	N
BRCA	TP53	17	7578271	7578271	Gain of NHLH1 binding site	Missense_Mutation	SNP	T	C	<0.05	DOWN	Y
HNSC	TP53	17	7578271	7578271	Gain of NHLH1 binding site	Missense_Mutation	SNP	T	A	<0.05	DOWN	Y
LGG	CIC	19	42791757	42791757	Gain of Nkx3-2 binding site	Missense_Mutation	SNP	C	T	<0.05	UP	Y
